# Insights into modifiable risk factors of retinal vascular occlusion: A Mendelian randomization study

**DOI:** 10.1097/MD.0000000000041752

**Published:** 2025-05-02

**Authors:** Bingcai Jiang, Xin Wei, Xiaochuan Cao, Changwei Zheng

**Affiliations:** a Department of Ophthalmology, Guizhou Provincial People’s Hospital, Guizhou, China; b Department of Ophthalmology, The People’s Hospital of Tongliang District, Chongqing, China.

**Keywords:** glaucoma, hypertension, Mendelian randomization, retinal vascular occlusion

## Abstract

Understanding the etiological risk factors for retinal vascular occlusion (RVO) is critical for prevention and treatment. While the effects of cardiovascular events, hypertension, glaucoma, obesity and glycemic risk factors on RVO are still controversial. This study employed two-sample Mendelian randomization (MR) analysis to investigate these causal risk factors. Single-nucleotide polymorphisms (SNPs) were used as instrumental variables (IVs). Genetic instruments for hypertension, glaucoma, obesity, cardiovascular events and glycemic risk factors were obtained from published genome-wide association studies (GWASs). Summary-level data for RVO and hypertension were obtained from the FinnGen consortium. MR analysis primarily utilized the inverse variance weighted (IVW) method, with MR-Egger and weighted median as supplementary approaches. Multivariable MR (MVMR) adjusting for hypertension or glaucoma of RVO were conducted. Heterogeneity was assessed using Cochrane’s Q test and *I*^2^, while MR-Egger intercept and MR-PRESSO tested horizontal pleiotropy. All MR analyses were performed within R software (4.1.3) using the R packages “TwoSampleMR” and “MR-PRESSO.” Genetic instruments for hypertension and glaucoma were significantly associated with RVO risk. A one-standard deviation (SD) increase in hypertension was associated with a higher risk of RVO [OR = 1.577, 95% CI = (1.342, 1.854), *P* < .001], while a one-SD increase in the log odds of genetically predicted glaucoma was associated with a higher risk of RVO [OR = 1.24, 95% CI = (1.115, 1.379), *P* < .001]. Meanwhile, hypertension and glaucoma were still significant in multivariable MR. There was not sufficient evidence to suggest cardiovascular events and obesity were associated with RVO risk. This MR study provided genetic evidence supporting that hypertension and glaucoma were causally associated with the risk of RVO. It may help guide clinical decisions in the management of RVO patients with hypertension and glaucoma.

## 1. Introduction:

Retinal vascular occlusion (RVO) is the most common vision-threatening disease. According to the site of occlusion, RVO can be classified as retinal vein occlusion and artery occlusion. The global prevalence of retinal vein occlusion is 0.77%, equivalent to an overall prevalence of 28.06 million, which leads to a heavy burden on society.^[1]^ The incidence of retinal artery occlusion is 2.58/100,000, which is much less than that of vein occlusion.^[2]^

Both retinal vein occlusion and artery occlusion will cause severe visual impairment. Some patients even lost vision because of serious complications, including vitreous hemorrhage, retinal detachment, and neovascular glaucoma. To date, no effective treatment is available, so determining the etiological risk factors for RVO may benefit clinical diagnosis and treatment.

However, the pathogenesis of RVO remains largely unknown. The etiology of RVO is multifactorial, involving genetic and environmental factors such as hypertension, glaucoma, cardiovascular events, obesity, and glycemic traits. While systemic diseases like hypertension and diabetes are recognized as potential risk factors, findings remain inconsistent, particularly for cardiovascular events, obesity, and glaucoma.^[3–6]^ Some studies have shown patients with cardiovascular events, including stroke, myocardial infarction, and atrial fibrillation, have an increased risk of RVO.^[7,8]^ Hayreh et al reported stroke has previously been identified as significantly associated with RVO.^[9]^ Roskal-Wałek et al argued that RVO patients are significantly more likely to experience stroke.^[10]^ In contrast, several studies based on direct evaluation did not show any high risk of RVO following cardiovascular events.^[2,11]^ Additionally, glaucoma is an important ocular risk factor for RVO, as reported in many studies.^[12–14]^

Although many studies on such relationships have been published, the results remain inconsistent. Some researchers have denied the accelerating effect of glaucoma on RVO risk.^[7,15]^ Moreover, findings on the effect of obesity on RVO remain inconsistent. An early study demonstrated that obesity was also involved in the development of RVO.^[16]^ However, a recent cohort study and meta-analysis suggested that it might not be related to RVO.^[1,7]^

It is well known that traditional retrospective studies are susceptible to confounding factors and reverse causality, which reduces their credibility.^[17]^ Thus, it is necessary to provide more evidence to disentangle the causal relationships between the risk factors and RVO. Katan first introduced the concept of Mendelian randomization (MR) in 1986.^[18]^ In recent years, MR analyses have been widely used for causal inference in epidemiology. The working principles of MR are similar to those of randomized clinical trials. Genetic variants depending on strongly associated single-nucleotide polymorphisms (SNPs) are used to randomly divide participants into different exposure/treatment levels instead of interventions in randomized clinical trials.^[19]^ It can reduce the bias caused by confounders or reverse causation.^[20]^ Recently, some MR studies indicated the high-density lipoprotein and diabetes may be an independent risk factor for^[21,22]^ RVO. This study employed two-sample MR to evaluate 11 risk factors for RVO, aiming to clarify their causal roles and corroborate previous findings.

## 2. Materials and methods

### 2.1. MR design and instrumental variable extraction

In this MR study, we extracted SNPs from genome-wide association studies (GWAS) as instrumental variables (IVs) for further analysis. SNPs were selected according to the following 3 basic criteria: SNPs were closely associated with exposure and reached genome-wide significance (*P* < 5 × 10^−8^); SNPs were not associated with any potential confounders and were independent of each other to avoid biases caused by linkage disequilibrium (*r*^2^ < 0.001, clumping distance = 10,000 kb); and SNPs are only linked to the outcome via the exposure.^[23]^

In univariable MR analysis (IVW, MR-Egger, weighted median), we simply tested the causation between each risk factor and RVO. IVW is an effective method to provide a consistent estimate of causality between exposure and outcome in the absence of horizontal pleiotropy under selected IVs are valid. Otherwise, if the IVs violate the premise of “no horizontal pleiotropy,” the IVW’s estimated results will be seriously biased.^[24]^ Given this, we conducted sensitivity analyses by using 2 supplementary MR methods: the weighted median method^[25]^ and MR-Egger.^[26]^ The weighted median assumes 50% of the weight is from valid SNPs. The MR Egger is similar to IVW, but it does not set the intercept term to zero, but rather estimates it during the calculation. In multivariable MR analysis, we included the significant risk factors from the univariable analysis and tried to identify the independent risk factors.

To evaluate the strengths of the remaining SNPs, an F statistic (F = beta^2^/se^2^; beta: beta for the SNP-exposure association, se: standard error) was calculated for each SNP.^[27]^ Then a mean F statistic for all SNPs was calculated, as shown in Table 1. Generally, an F statistic > 10 indicates that no obvious bias is caused by weak IVs. SNPs with less statistical power were removed (F statistic < 10). MR-Steiger filtering was used to remove variations that were more strongly correlated with RVO than with risk factors.^[28]^ Full details of the SNPs and MR-Steiger are provided in the Datasets S1–S11, Supplemental Digital Content, https://links.lww.com/MD/O794; https://links.lww.com/MD/O795; https://links.lww.com/MD/O796; https://links.lww.com/MD/O797; https://links.lww.com/MD/O798; https://links.lww.com/MD/O799; https://links.lww.com/MD/O800; https://links.lww.com/MD/O801; https://links.lww.com/MD/O802; https://links.lww.com/MD/O803; https://links.lww.com/MD/O804. This study was performed using publicly available GWAS summary statistics, and ethical approval was obtained in all original studies.

**Table 1:** Basic information of the genome-wide association studies used in this study.

Exposures	No. of SNPs	Unit	Sample size	F (mean)	PMID
Hypertension	40	1 unit in logOR	205,694	48.87	–
Type 2 diabetes	114	1 unit in logOR	659,316	76.72	30054458
Fasting glucose	62	SD	200,622	139.78	34059833
Fasting insulin	38	SD	151,013	51.8	34059833
HbA1c	69	SD	146,806	108.87	34059833
Stroke	17	1 unit in logOR	446,696	40.46	29531354
Myocardial infarction	24	1 unit in logOR	171,875	61.78	26343387
Atrial fibrillation	108	1 unit in logOR	1030,836	96.11	30061737
Glaucoma	75	1 unit in logOR	351,696	56.91	31959993
Waist-to-hip ratio	27	SD	212,244	45.56	25673412
Body mass index	141	SD	315,347	56.3	30108127

－ = unpublished, F = F statistics, logOR = logarithm of OR, No. of SNPs = number of single nucleotide polymorphisms, PMID = ID of publication in PubMed.

### 2.2. Data sources

Eleven risk factors were analyzed: hypertension, type 2 diabetes (T2DM), fasting glucose, fasting insulin, glycated hemoglobin (HbA1c), stroke, myocardial infarction, atrial fibrillation, body mass index (BMI), waist-to-hip ratio (WHR), and glaucoma. All datasets are publicly available online from IEU Open GWAS Project database. To avoid the effects of population stratification, all variants and their associated summary-level datasets were only extracted from studies with participants of European descent. We extracted IVs of BMI from a large multiethnic genome-wide association study, with a total sample size of 315,347 from individuals of European descent.^[29]^ For waist-to-hip ratio GWAS from the GIANT (Genetic Investigation of ANthropometric Traits) consortium, we only used the summary statistics from 212,244 European participants.^[30]^ The IVs of T2DM were extracted from a meta-analysis of GWAS including 62,892 cases and 596,424 controls.^[31]^ The GWAS summary statistics of other glycemic traits were from a meta-analyses of glucose and insulin-related traits consortium.^[32]^ We only used the European summary statistics with a sample size of 200,622 for fasting glucose, a sample size of 151,013 for fasting insulin and a sample size of 146,806 for HbA1c. The IVs of stroke were extracted from a meta-analysis of GWAS including 40,585 cases and 406,111 controls.^[33]^ The GWAS summary statistics of myocardial infarction included 43,676 cases and 128,199 controls.^[34]^ For atrial fibrillation, the GWAS study included 60,620 cases and 970,216 controls.^[35]^ For glaucoma, the GWAS included 133,492 cases and 90,939 controls.^[36]^ For hypertension and RVO, the GWAS was from the FinnGen biobank. Participants with RVO included 1595 cases and 203,108 controls of individuals, and participants with hypertension included 42,857 cases and 162,837 controls. The FinnGen study is a national public health project in Finland.^[37]^ RVO status was diagnosed according to the ICD codes. All studies adjusted for study-specific components. The information of the genetic datasets used in this study was summarized in Table 1.

### 2.3. Statistical analyses

IVW, MR–Egger and weighted median were employed to estimate the causal effects in the two-sample MR analysis. Heterogeneity of IVs was tested using Cochrane’s Q test and *I*^2^*. P* < .05 of Cochrane’s Q indicated the existence of heterogeneity.^[38]^ An *I*^2^ value > 25% was regarded as significant heterogeneity.^[39]^ Pleiotropy was assessed with MR-Egger intercept^[40]^ and MR-PRESSO.^[41]^ *P* < .05 of the MR-Egger intercept and MR-PRESSO Global test suggested significant horizontal pleiotropy. Using a conservative approach, a Bonferroni-corrected significance level of *P* < .004 (0.05 divided by 11) was considered to be suggestive of causality. All MR analyses were performed using R software (4.1.2) and the R packages “TwoSampleMR” and “MR-PRESSO.”

## 3. Results

The number of SNPs ranged from 17 to 141 (Table 1). The F statistics were all greater than the empirical threshold of 10, suggesting that all SNPs had sufficient validity, and the mean F statistics in each subgroup are shown in Table 2.

**Table 2:** Mendelian randomization results of heterogeneity and pleiotropy on RVO.

Exposures	No. of SNPs	*I* ^2^	Cochrane’s Q statistic		MR-Egger test		MR-PRESSO
			Q	*P* value	Intercept	*P* value	*P* value
Hypertension	40	0%	36.793	.571	0.02	.413	.603
Type 2 diabetes	114	0%	112.407	.498	0.007	.439	.503
Fasting glucose	62	0%	48.141	.884	−0.01	.293	.896
Fasting insulin	38	0%	35.265	.551	0.0001	.995	.541
HbA1c	69	0%	64.76	.588	−0.006	.538	.581
Stroke	17	35.91%	24.965	.07	0.058	.479	.083
Myocardial infarction	24	7.39%	24.835	.359	−0.032	.172	.369
Atrial fibrillation	108	22.83%	138.645	.021	0.009	.334	.027
Glaucoma	75	6.03%	78.752	.331	−0.011	.487	.335
Waist-to-hip ratio	27	0%	18.814	.844	0.02	.581	.854
Body mass index	141	1.21%	141.709	.443	−0.01	.289	.449

### 3.1. Causal effect of hypertension on RVO

**Figure 1.**
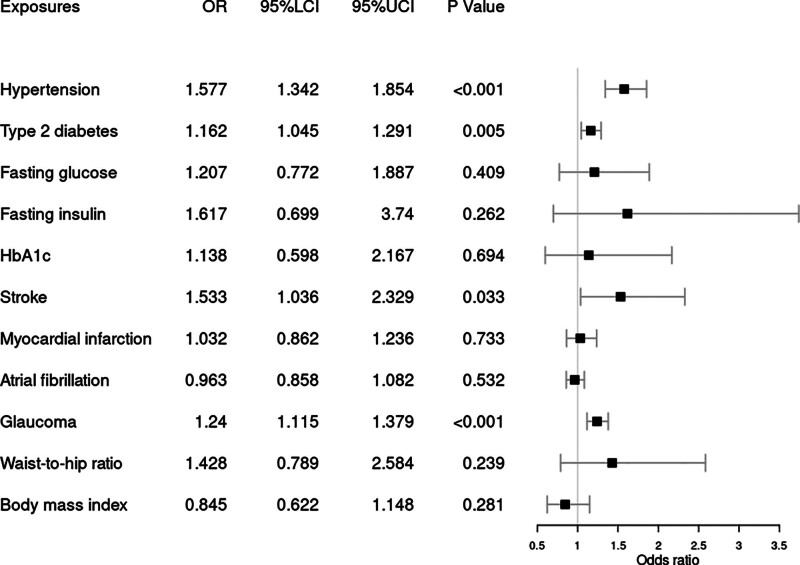
Forest plot of Mendelian randomization results from etiological risk factors. 95% LCI = lower limit of 95% CI, 95% UCI = upper limit of 95% CI.

**Figure 2.**
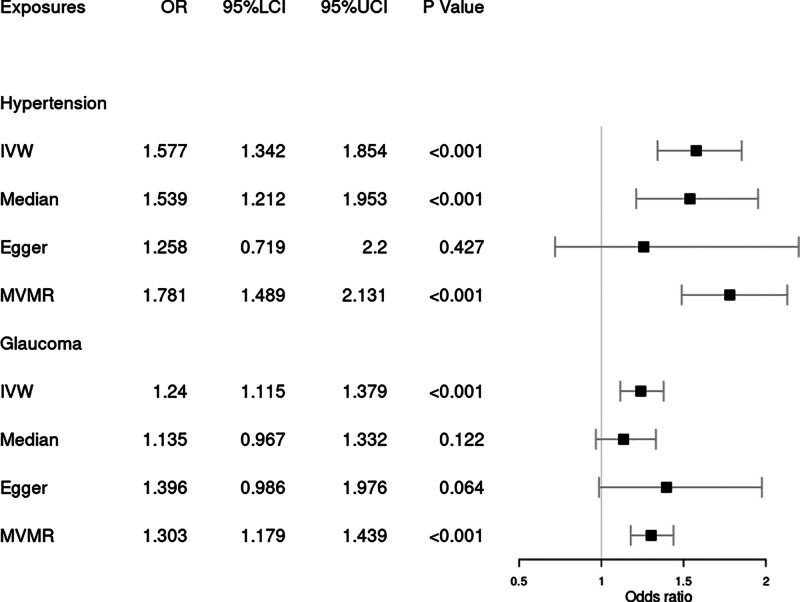
Forest plot of Mendelian randomization results from hypertension and glaucoma. Egger = MR–Egger, IVW = inverse variance weighted, Median = weighted-median, MVMR = multivariable MR, 95% LCI = lower limit of 95% CI, 95% UCI = upper limit of 95% CI.

In the MR analysis of the relationship between hypertension and RVO, IVW method suggested a causal association between them. A one-SD increase in hypertension was associated with an increased odds ratio (OR) of RVO [OR = 1.577, 95% CI = (1.342, 1.854), *P* < .001] based on the IVW method (Fig. 1). This was supported by the weighted median analysis [OR = 1.539, 95% CI = (1.212, 1.953), *P* < .001] and multivariable Mendelian randomization (MVMR) analysis [OR = 1.781, 95% CI = (1.489, 2.131), *P* < .001], as shown in Figure 2. There was no heterogeneity according to Cochrane’s Q test (Q = 36.793; *P* = .571) or *I*^2^ (*I*^2^ = 0%) and no directional pleiotropy according to the MR–Egger test (intercept = 0.02; *P* = .413) or MR-PRESSO Global test (*P* = .603), as shown in Table 2. Using the MR-Steiger test, none of the variants were removed and results remained unchanged.

### 3.2. Causal effect of glaucoma on RVO

Genetic predisposition to glaucoma was also associated with the overall risk of RVO based on the IVW method [OR = 1.24, 95% CI = (1.115, 1.379), *P* < .001] (Fig. 1). This was supported by the MVMR analysis [OR = 1.303, 95% CI = (1.179, 1.439), *P* < .001] (Fig. 2). No heterogeneity was detected by Cochrane’s test (Q = 78.752, *P* = .331) or *I*^2^ (*I*^2^ = 6.03%) (Table 2). No evidence was found of directional pleiotropy using MR–Egger test (intercept = −0.011; *P* = .487) or MR-PRESSO (*P* = .335), as shown in Table 2. No one SNP was excluded by MR-Steiger.

### 3.3. Causal effect of other factors on RVO

Weak evidence suggested a possible association between T2DM and RVO [OR = 1.162, 95% CI = (1.045, 1.291), *P* = .005] by IVW. However, it not reach the Bonferroni-corrected significance level (*P* < .004). There was no evidence to support a causal relationship between BMI, WHR, fasting glucose, fasting insulin, HbA1c, stroke, myocardial infarction, or atrial fibrillation and RVO risk (Table 3).

**Table 3:** Mendelian randomization results of inverse variance weighted, weighted median and MR-Egger methods.

Exposures	No. of SNPs	Inverse varianse weighted				Weighted median				MR-Egger			
		OR	95% LCI	95% UCI	*P* value	OR	95% LCI	95% UCI	*P* value	OR	95%LCI	95% UCI	*P* value
Hypertension	40	1.577	1.342	1.854	<.001*	1.539	1.212	1.953	<.001†	1.258	0.719	2.2	.427
Type 2 diabetes	114	1.162	1.045	1.291	.005	1.058	0.878	1.275	.556	1.061	0.825	1.365	.644
Fasting glucose	62	1.207	0.772	1.887	.409	1.333	0.685	2.593	.398	1.732	0.775	3.869	.185
Fasting insulin	38	1.617	0.699	3.74	.262	1.945	0.58	6.515	.281	1.603	0.11	23.291	.732
HbA1c	69	1.138	0.598	2.167	.694	1.937	0.699	5.36	.661	1.595	0.458	5.555	.468
Stroke	17	1.533	1.036	2.329	.033	1.553	0.958	2.518	.074	0.586	0.04	8.425	.7
Myocardial infarction	24	1.032	0.862	1.236	.733	1.032	0.796	1.336	.813	1.383	0.888	2.153	.166
Atrial fibrillation	108	0.963	0.858	1.082	.532	0.909	0.753	1.098	.323	0.909	0.753	1.098	.252
Glaucoma	75	1.24	1.115	1.379	<.001‡	1.135	0.967	1.332	.122	1.396	0.986	1.976	.064
Waist-to-hip ratio	27	1.428	0.789	2.584	.239	1.151	0.481	2.751	.752	0.656	0.04	10.685	.769
Body mass index	141	0.845	0.622	1.148	.281	0.782	0.489	1.252	.306	1.33	0.546	3.238	.531

* *P* = 3.31e-08.

† *P* = 3.94e-04.

‡ *P* = 7.39e-05.

## 4. Discussion

This study explored potential causal associations between the risk of RVO and the predominant risk factors such as hypertension, glycemic traits, obesity, glaucoma, and cardiovascular events using MR analysis. Among these factors, hypertension was causally associated with the risk of RVO, which was consistent with previous findings. Meanwhile, this MR study also supported the causal effect of glaucoma on RVO. Furthermore, MVMR analyses indicated that glaucoma or hypertension might be an independent risk factor for RVO.

Hypertension is associated with multiple systemic effects, including the eye. A number of conventional observational studies and meta-analyses have consistently implicated hypertension as a risk factor for developing RVO.^[1,42]^ Song’s meta-analysis demonstrated hypertension was the strongest risk factor for any retinal vein occlusion, with a meta- odds ratio of 2.82 (95% CI = 2.12, 3.75).^[1]^ The present MR found further genetic evidence to demonstrate that hypertension is a potential risk factor for RVO [OR = 1.539, 95% CI = (1.212, 1.953)]. Although the exact pathogenesis of RVO remains unknown, it likely includes hypercoagulability, endothelial injury and hemodynamic changes, which is known as Virchow’s triad.^[43]^ Many studies have also shown endothelial injury and histological changes in vessel walls in patients with RVO.^[44–46]^ Hypertension can lead to vascular changes, including retinal arteriolar narrowing and arteriovenous nicking, which are easily visible in the retina. In addition, the luminal surface of damaged endothelial cells is less able to prevent thrombus formation after minor injury caused by turbulent blood flow. A study reported that 90% of patients with retinal vein occlusion showed signs of intima-medial layer hypertrophy and intravenous thrombosis.^[47]^ Retinal emboli may be found in the vessels in up to 20% of central retinal vein occlusions and up to 70% of branch retinal artery occlusions.^[48,49]^ Therefore, hypertension-induced vascular wall degeneration may be an important factor in the occurrence of RVO. This strong and consistent link between hypertension and RVO suggests the benefits of blood pressure management in the prevention of RVO.

Glaucoma is a global disease that causes irreversible vision loss. Numerous studies on the causal relationship between glaucoma and RVO have been published.^[12,14,50,51]^ A recently meta-analysis demonstrated that OR of glaucoma as a risk factor for RVO was 4.01 [95% CI = (3.28, 4.91)].^[51]^ Using MR techniques, we found genetic evidence to indicate that glaucoma is causally associated with the risk of RVO [OR = 1.24, 95% CI = (1.115, 1.379)]. A vascular hypothesis of glaucoma might explain the association between glaucoma and RVO.^[52]^ Elevated intraocular pressure may compress vessel walls, leading to subsequent blood vein intimal proliferation.^[53]^ Gao et al indicated that glaucoma patients have narrower retinal arteries and veins than control groups.^[54]^ Furthermore, researchers have already found that glaucoma precedes vascular occlusion.^[55]^ In fact, disc hemorrhage representing small vein occlusions is frequently seen in patients with glaucoma in the clinic. Therefore, the vascular etiology of glaucoma may facilitate the development of RVO. In addition, displacement of the lamina cribrosa caused by high intraocular pressure can change the shape and course of the central retinal vein.^[56]^ It can increase turbulence and endothelial stress, which may also increase the risk of RVO in eyes with glaucoma. So, glaucoma should be kept in mind when investigating patients with RVO in the clinic.

The role of diabetes mellitus remains controversial in the literature. Some studies have shown that RVO is associated with T2DM,^[57,58]^ whereas others have shown no association.^[59]^ An Indian facility-based opportunistic survey assessing the prevalence of RVO in diabetic patients reported that 3.4% of T2DM patients developed RVO. Meanwhile, it noted that the duration of T2DM apparently had no influence on the occurrence of RVO.^[60]^ In a Japanese cross-sectional study, Yasuda et al found that T2DM was not associated with RVO risk after adjusting for age and sex.^[61]^ It’s well known that cohort studies were not possible to rule out underlying confounders, such as the serum lipids which was a risk factor for both T2DM^[62]^ and RVO,^[21]^ thus complicating their causal relationship. Recently, two MR studies suggested a significant causal association between T2DM and retinal artery occlusion and retinal vein occlusion.^[22,63]^ Similarly, we found weak evidence that a higher genetic liability to T2DM increases the risk of RVO. But the other glycemic traits including fasting glucose, fasting insulin, HbA1c, are not causally related RVO. The underlying molecular mechanisms by which T2DM promotes RVO development may related to inflammatory mediators and oxidative stress, which is a topic deserving of further research.^[64,65]^

Except for hypertension, glaucoma and glycemic risk factors, the effects of cardiovascular events, including stroke, myocardial infarction and atrial fibrillation, on RVO are still uncertain. The association of RVO with cardiovascular events is clinically relevant, as the pathophysiological background might be atherosclerosis, a thrombotic event, or a combination of both. Previous studies revealed a positive association between stroke,^[4,5]^ myocardial infarction,^[66,67]^ atrial fibrillation^[4,7]^ and RVO. But a recently bidirectional MR study found that no significant causal effect of stroke and myocardial infarction on RVO.^[68]^ The present MR further included the atrial fibrillation. The MR estimates for the 3 cardiovascular events analyzed in this study also tended to support the null association between them. The relatively small number of patients with retinal vascular occlusion in the FinnGen consortium may account for this null association. Besides, the previously observed associations or cohort studies are prone to confounding factors, and these findings are limited by uncontrolled confounding factors, as Rim et al pointed out.^[69]^ More studies are warranted to identify these potential confounders and elucidate their complex relationship. Finally, the association between obesity and RVO is still unsettled, as the association was either positive^[3]^ or null.^[6,7]^ Obesity is widely accepted risk factor for many diseases, such as cardiovascular diseases, hypertension and diabetes mellitus.^[70,71]^ In the MR study obesity including BMI and WHR was not causally associated with RVO.

This study clarified the causal associations between 11 etiological risk factors and RVO using the MR method. This study has several advantages. First, our study is the MR design, which mitigates bias from reverse causation and confounding. Second, our MR study primarily utilized European subjects, thus minimizing bias due to population stratification. However, several limitations also exist in this study. We used the summary statistics of RVO from FinnGen round 5. These included RVO patients were not divided into two main sub-entities, retinal vein occlusion and artery occlusion. Therefore, it is uncertain whether the risk factors causally related with a specific type of RVO. Moreover, the greatest concern in MR studies is horizontal pleiotropy, which occurs when genetic variants influence the outcome of more than one pathway.^[38]^ We used two main means to detect horizontal pleiotropy, including the MR–Egger intercept and MR-PRESSO methods. In addition, MR-Steiger test was used to filter all selected SNPs, hoping to minimize the bias. However, it is not possible to completely rule out the presence of residual pleiotropy. Last but not least, care should be taken when expanding our conclusions to other populations, as the present MR analysis utilized subjects of primarily Europeans.

In conclusion, the present study demonstrates that exposures of hypertension and glaucoma may increase the risk of RVO. It may help guide clinical decisions in the management of RVO patients with hypertension and glaucoma.

## Author contributions

**Conceptualization:** Changwei Zheng, Bingcai Jiang, Xin Wei.

**Data curation:** Changwei Zheng, Bingcai Jiang, Xin Wei.

**Formal analysis:** Changwei Zheng, Bingcai Jiang, Xin Wei, Xiaochuan Cao.

**Funding acquisition:** Changwei Zheng, Bingcai Jiang, Xin Wei.

**Investigation:** Changwei Zheng, Bingcai Jiang.

**Methodology:** Changwei Zheng, Bingcai Jiang, Xin Wei.

**Project administration:** Changwei Zheng, Bingcai Jiang, Xin Wei.

**Resources:** Changwei Zheng, Bingcai Jiang, Xin Wei.

**Software:** Changwei Zheng, Bingcai Jiang.

**Supervision:** Changwei Zheng, Bingcai Jiang, Xin Wei.

**Validation:** Changwei Zheng, Bingcai Jiang, Xin Wei.

**Visualization:** Changwei Zheng, Bingcai Jiang, Xin Wei.

**Writing – original draft:** Changwei Zheng, Bingcai Jiang, Xin Wei.

**Writing – review & editing:** Changwei Zheng, Bingcai Jiang, Xin Wei.

## Supplementary Material


